# The efficacy of convenient cleaning methods applicable for customized abutments: an in vitro study

**DOI:** 10.1186/s12903-021-01436-z

**Published:** 2021-02-18

**Authors:** Sunjai Kim, Changhun Choi, Yunsu Cha, Jae-Seung Chang

**Affiliations:** 1 Department of Prosthodontics, Gangnam Severance Dental Hospital, College of Dentistry, Yonsei University, Seoul, Korea; 2 Department of Prosthodontics, College of Dentistry, Yonsei University, Seoul, Korea; 3 College of Human Ecology, Seoul National University, Seoul, Korea; 4 Department of Prosthodontics, Gangnam Severance Dental Hospital, College of Dentistry, Yonsei University, 211 Eonju-ro, Gangnam-gu, Seoul, 06273 Korea

**Keywords:** CAD/CAM, Chlorhexidine, Bacteria, Implants, Foreign bodies, Titanium, Sterilization

## Abstract

**Background:**

The demand for implant dentistry, which includes customized abutments, is increasing. A lot of pollutions are generated on the customized abutment surface following milling procedure. This study evaluated the surface topography and cleanliness of customized abutments after cleaning procedures, which are simply applicable in the dental clinic.

**Methods:**

Thirty computer-aided design and computer-aided manufacturing internal connection type titanium abutments were produced, milled, and randomly divided into 3 groups: steam cleaning (control group), chlorhexidine (CHX) scrubbing (test group 1), and ultrasonic cleaning with CHX solution, acetone, and ethyl alcohol (test group 2). Each group was evaluated using microscopic and microbial analysis.

**Results:**

Foreign bodies were observed on the abutment surfaces in control group and test group 1, but not in test group 2. Bacteria were observed on 40% of the agar plates following steam cleaning; most of the colonies consisted of Bacillus cereus and Staphylococcus warneri. Colony growth was absent following test group 1 and 2.

**Conclusion:**

For customized abutments, cleaning with steam is ineffective. CHX scrubbing effectively eliminates only bacteria. Ultrasonic cleaning with CHX solution, acetone, and ethyl alcohol successfully removes both foreign bodies and bacteria. Thus, the ultrasonic cleaning method is conveniently applicable in the dental clinic for eliminating contamination of the customized abutment surface.

## Background

The health of the peri-implant soft tissue, which is located between the oral tissue and bone, acts as a significant role in implant’s long-term success. The soft tissues that surround the implant are hypovascular and hypocellular wound tissues with a significantly lower immunological capacity than the periodontal tissues. Thus, peri-implant soft tissue has a lower resistance to infection by bacterial colonies [1, 2]. Furthermore, mechanical overload and prosthodontic processes can also exert a negative effect on these tissues [3, 4]. The upper structure of an implant, known as an abutment, is in direct contact with the surrounding tissues and can therefore affect the health and shape of the peri-implant soft tissues. Thus, the material, surface characteristics, and surface treatments become important factors in determining the degree of oral health associated with dental implants. The chemical and structural features of abutments also affect the surrounding bone and connective tissues. Furthermore, abutments also play an important role in bioaffinity as they are exposed to the oral cavity through the gingival mucosa. The presence of contaminants in the implant-abutment interface initiates an inflammatory response, which may damage tissue and activate osteoclasts, thus subsequently resulting in bone resorption and the resetting of biologic width [5, 6].

Due to a recent demand for aesthetic dental implants, customized abutments usage has been significantly increased. Despite this, the importance of abutment surface treatments has been generally overlooked in applying to patients. There is insufficient information regarding manufacturers and techniques for surface treatment and sterilization. Canullo et al. [7] confirmed the presence of pollutions on the abutment surface following traditional milling procedure. SEM analysis revealed that micro-particles covered 0.025% of the milled abutment surface, while no micro‐particles and pollution were revealed on the whole abutment surface before milling process. Sawase et al. [8] have highlighted many differences between manufacturers with respect to surface topography, composition, milling and finishing procedures, and cleaning and sterilizing procedures of customized abutments. After manufacture, customized abutments must undergo steam-cleaning to remove any contaminants located on both the internal and external surfaces [9]. The presence of contaminants that remain even after steam-cleaning can cause inflammatory responses in peri-implant tissues and form plaques and bacterial colonies, which can result in infection on the collar surface of implants and abutments. The removal of such microbial contaminants reduces bacterial adhesion and osteoclast activation. The contaminants were demonstrated to affect implant–abutment fit, leading to an increased mechanical stress on connection. This condition may induce preload loss or fracture and cause biological complications due to bacterial penetration within a possible fixture–abutment gap [10]. Surface contaminants on abutments can be removed by steam-cleaning, but such laboratory procedures cannot completely eliminate the contaminants.

CHX, which is in routine clinical use, has been demonstrated as one of the most effective chemical plaque-control compounds [11–14]. It is a widely used antiseptic with beneficial antimicrobial effects on Gram-positive and Gram-negative bacteria and on fungi as well [15]. Furthermore, it has shown to be effective that 0.12% CHX solution mouthwash is able to remove the severe acute respiratory syndrome coronavirus 2 (SARS-CoV-2) [16].

Since CHX is a positively charged bisbiguanide, it is helpful at adsorbing different sites that are negatively charged, such as titanium surfaces, salivary pellicle on teeth, and mucous membranes as the biofilm on the tooth surfaces [17–20]. In this context, CHX scrubbing can deserve potential benefit, but there is still no sufficient evidence that CHX scrubbing alone could significantly remove bacteria and foreign bodies on customized abutments in human.

Consequently, additional procedures, such as ultrasonic cleaning, which mechanically remove contaminants with high frequency waves in aqueous or organic medium, are recommended prior to clinical application [21, 22]. Canullo et al. [23, 24] compared a group of customized abutments that was steam-cleaned for 5 s with a group that was subjected to argon plasma cleaning/sterilization. Two-year follow-up results showed that peri-implant bone resorption was lower for the group of patients whose implants had been cleaned and sterilized. However, dental clinics are rarely equipped with this type of sterilizer, so there is a limitation on treating the surface of customized abutments.

This article performed a study to evaluate the surface topography and cleanliness of customized abutments after convenient cleaning procedures applicable for customized abutment in the dental clinic.

## Methods

### Customized titanium abutments

Thirty CAD/CAM customized titanium abutments were fabricated with an internal connection type. All abutments were manufactured in a commercial dental laboratory. Milling and polishing were performed using carbide burs mounted on a miller (ARUM5X-100, Doowon electronics, Daejun, Korea). Following the milling process, abutments were randomly divided into 3 groups of 10.

### Cleaning methods

**Fig. 1:**
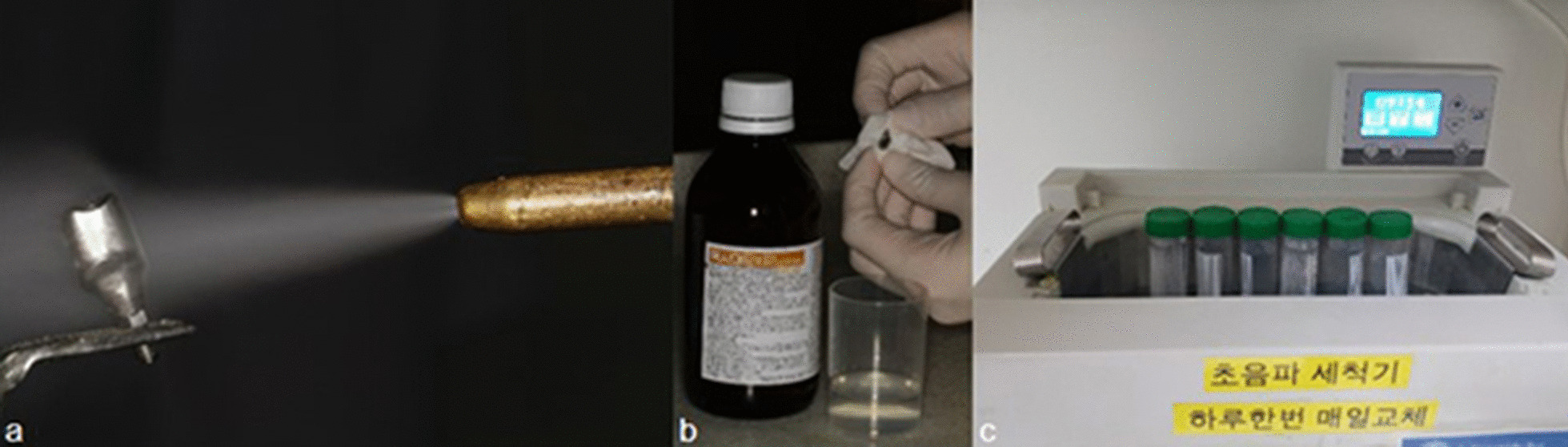
Customized titanium abutments cleaned with three different methods: **a** steam cleaning, **b** CHX scrubbing, and **c** ultrasonic cleaning

The abutments assigned to control group were steam cleaned at 4 MPa and 150 °C for 10 s using a steam cleaner (Steam Man Plus, Shin Myung Dental Co., Seoul, Korea). The abutments assigned to test group 1 were steam cleaned, after which they were scrubbed 5 times with gauze soaked in 0.12% CHX (Hexamedine, BUKWANG PHARM. Co., Ltd., Seoul, Korea) as part of the conventional protocol and were soaked in distilled water for 5 min. Lastly, the abutments assigned to test group 2 were sequentially immersed in three different solutions, which are 0.12% CHX solution, pure acetone, and 98% ethyl alcohol, at 50 °C with an ultrasonic cleaner (UC125 Ultrasonic Cleaning System, Coltene/Whaledent INC, USA) for 10 min each. After immersion in each solution, samples were soaked in distilled water for 5 min. The total cleaning time was 45 min (Fig. 1).

### Microscopic and Microbiological analysis

The abutments were inserted in the surface analyzing device after steam cleaning, CHX scrubbing, and ultrasonic cleaning Scanning electron microscopy (SEM) (S-3000 N, Hitachi Ltd., Tokyo, Japan) was used to scan the connection area and the hexagon area of the abutments.

**Fig. 2:**
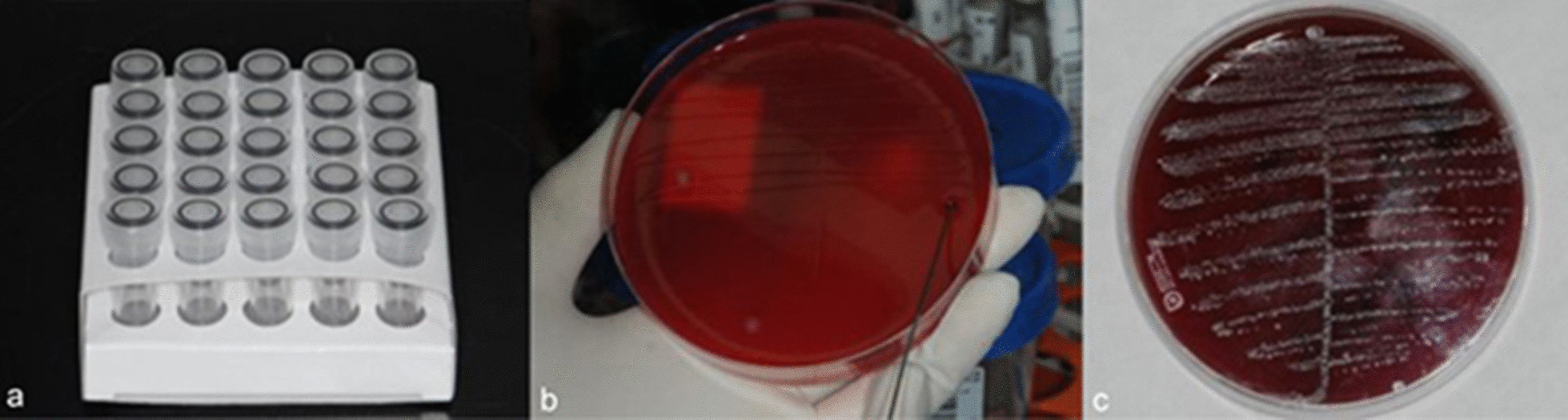
Microbiological analysis of customized titanium abutments. **a** Incubation in a thioglycolate microtube. **b** Culture on an agar plate. **c** Observation of colonies

To evaluate the bacterial contamination on the sample abutments, they were transferred to sterilized 1.5 ml micro-tubes containing 1 ml of thioglycolate medium and incubated overnight under aerobic conditions. The bacterial colonies on which blood agar plates were incubated in 35℃ aerobic condition for 24 h, were observed (Fig. 2).

### Statistical analysis

Statistical analysis was performed using SPSS version 23.0 statistical software (SPSS Inc., Chicago, IL, USA). The Fisher exact set was performed in order to compare the frequency of bacterial growth in each group and the level of significance was set as 0.05. Additionally, bacterial taxonomy was analyzed using matrix assisted laser desorption ionization time of flight mass spectrometer (MALDI TOF MS) (Microflex LT, Bruker Elemental GmbH, Kalkar, Germany).

## Results

**Fig. 3:**
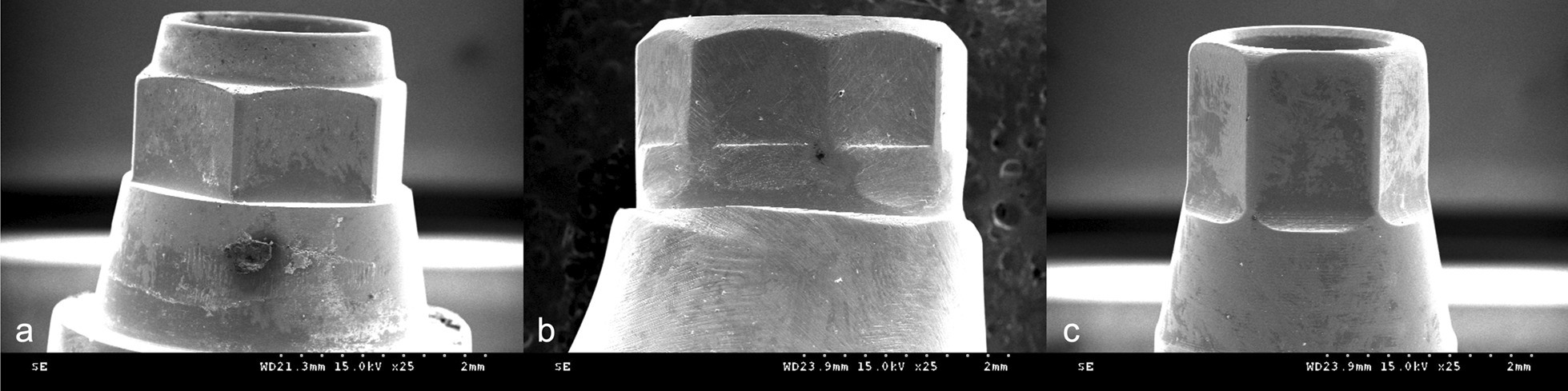
Scanning electron micrographs of abutments after different cleaning methods (× 25 magnification): **a** steam cleaning, **b** CHX scrubbing, and **c** ultrasonic cleaning

**Fig. 4:**
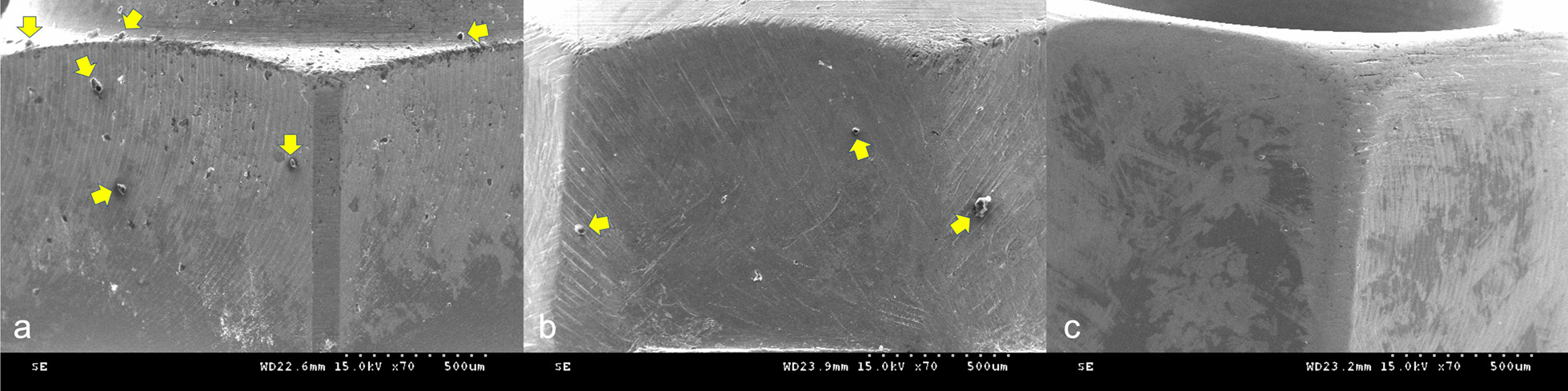
Scanning electron micrograph of contaminants (indicated with arrows) on the abutment surface (× 70 magnification): **a** steam cleaning, **b** CHX scrubbing, and **c** ultrasonic cleaning

Foreign bodies were observed in control group and test group 1, but not in test group 1 (Fig. 3). Arrows indicate foreign bodies on the abutment surfaces connected with implant and the average area of contamination across the two groups was 50 μm (Fig. 4).

Bacterial colonies were found on 40% of the agar plates in control group, but not in test group 1and 2. A statistical comparison of the different cleaning groups revealed a higher frequency of bacterial colonies in the steaming group than in the CHX scrubbing and ultrasonic cleaning groups (Table 1). The majority of the colonies consisted of Bacillus cereus and Staphylococcus warneri detected by MALDI-TOF MS analysis.

**Table 1:** Comparison of bacterial colonies after different cleaning methods

Cleaning methods				
	Control	CHX scrubbing	Ultrasonic	Total
Colonies	4	0	0	4
Without Colonies	6	10	10	26
P value^a^	0.023*			

^a^Fisher exact test

*P < 0.05 is considered statistically significant

## Discussion

Although grinding down the transmucosal surface of customized abutments to ensure softness can prevent initial attachment of soft tissues, a very rough abutment surface can increase plaque adhesion. In other words, subgingival and supragingival plaque formation is delayed when the surface roughness or Sa-value of abutments is lower than 0.2 μm, but soft tissue attachment is disrupted when the surface is too smooth. Hence, implant abutments with a Sa-value of 0.2 μm have a balanced response toward bacterial adhesion and soft tissue stability; thus, clinically, Sa-values between 0.15 and 0.25 μm are recommended for better soft tissue stability and lesser bacterial adhesion [25, 26]. In this study, a microscopic characterization of the surface of customized abutments was performed after processing, which revealed the presence of substantial contaminants such as lubricant microparticles and titanium fragments with an average size of 50 μm. Ultrasonic cleaning was also found to remove more contaminants than did steam cleaning or CHX scrubbing following steam cleaning.

One of the most important combinations requisite of an implant’s prosthetic stability is the interface between the implant and abutment, as even small movements or misalignments in this interface can loosen abutment screws, cause abutment fracture, and affect soft tissues [27–29]. The stability of the implant-abutment interface correlates with the connection type and fatigue characteristics of the screw and the precision of the implant components. Ideal preload is achieved by precise connections between implant components; imprecise connections caused by debris or contaminants on the implant-abutment interface or screw decreases the preload and prevents ideal connections [30, 31]. In this study, the average 50 μm foreign bodies of the abutment surface connected with implant can also negatively affect the stability of the interface between the implant and abutment. According to Olefjord and Hansson [32], the sulfur, phosphor, and silicate particles that have been observed on the surface of an abutment are caused by the laboratory process, and negatively affect the soft tissue around implants [33]. These particles can cause osteoclasis and can induce monocyte/macrophage survival in vitro through an endogenous mediator [33]. Titanium particles were shown to induce acute inflammation via increases in interleukin (IL)-1β secretion and IL-1-associated signaling by promoting the NALP3 inflammasome, which is time-limiting enzymes of PGE2 synthesis, and RANL/RANKL, which differentiates osteoclasts [34, 35]. Therefore, these particles cause bone resorption by enhancing osteoclast differentiation. The occurrence of this phenomenon in the connection area of the abutment increases macrophages and osteoclasts in the connective tissue. Thus, the cancellous bone will cause local inflammation, resulting in the reset of biological width and bone resorption.

This study aimed to evaluate the surface topography and cleanliness of customized abutments after convenient cleaning procedures in the clinic. Cleaning only with steam resulted in residual foreign bodies on abutment surface, and bacterial colonies were found on 40% of the agar plates. Thus, steam cleaning is insufficient for bacterial decontamination. Bacillus cereus and Staphylococcus warneri, both of which were found in this study, are opportunistic pathogens. Gram-positive anaerobic bacteria Bacillus cereus proliferates in various environments, such as soil and water, with an optimal growth temperature of 28–35℃; these bacteria can cause emetic and diarrheal food poisoning. Gram-positive anaerobic bacteria Staphylococcus warneri, which have an optimal growth temperature of 30–40℃, can cause bacteremia.

Recently, 0.12% CHX solution mouthwash can effectively reduce the SARS-CoV-2 for a short period of time [16]. The viral load of SARS-CoV-2 was high in saliva, but it decreased for 2 h after 30 s of CHX mouthwash [16]. Due to this effectiveness, various international association have recommended the use of a pre-procedural mouth rinse to obviate the danger of SARS-CoV-2 transmission throughout dental treatment. For example, the New Zealand Dental Association highly suggests 0.2% CHX for 30 s for a pre-procedure and when this is not applicable, the alternative way can be used, which is a swab soaked in 1% CHX [36]. CHX, which is used extensively in dentistry, is a broad-spectrum antimicrobial agent [37]. In this study, the CHX solution that is effective for reducing the SARS-CoV-2 was also involved in both CHX scrubbing and ultrasonic cleaning methods, and when those methods were applied to customized abutments, it provided antimicrobial effect.

However, as a few studies suggested, its benefits are limited. According to Chin et al. [38], CHX has bactericidal capacity but does not actually remove biofilm from the implant surface. In this present study, it also reveals that CHX scrubbing is insufficient to remove foreign body on customized abutments.

Additionally, foreign bodies and bacterial growth were absent on the abutments after ultrasonic cleaning. Therefore, ultrasonic cleaning, which can remove both foreign bodies and bacteria, is generally used in most dental clinics, so it is thought to be a convenient way for the surface treatment of customized abutments without special disinfection procedures and sterilizers. Among three different solutions with an ultrasonic cleaner, 0.12% CHX solution and 98% ethyl alcohol acted as an antiseptic and disinfection and the acetone was used as organic solvent to remove lubricant and titanium smear layer during the milling procedure. However, since customized abutments were sequentially immersed in three different solutions with ultrasound, the physical effects of ultrasonic cleaner and the effect of antimicrobial solutions were not separately evaluated in limitation of this study. The further studies will be necessary to assess each effect of antimicrobial solutions and ultrasonic procedure.

There is no consensus regarding the cleaning protocol applicable to customized abutments. In addition, the majority of the universities and hospitals do not have an established set of standards [39]. Therefore, it is essential to develop standardized cleaning and decontamination methods to remove contamination of customized abutments, which can negatively affect the biological stability of peri-implant tissues in the long term.

## Conclusions

For the clinical use of customized abutments, steam cleaning is ineffective, CHX scrubbing is effective in removing bacteria, and ultrasonic cleaning with CHX solution, acetone, and ethyl alcohol is very effective in removing both foreign bodies and bacteria from the surface of the abutments. Therefore, ultrasonic cleaning is an effective procedure that can be in used dental clinics as it can be conveniently applied for abutments.

## Data Availability

The data that support the findings of this study were presented in detail (Table 1).

## Abbreviations

CHX: Chlorhexidine
CAD/CAM: Computer-aided design and computer-aided manufacturing

## References

[1] Berglundh T, Lindhe J, Jonsson K, Ericsson I (1994). The topography of the vascular systems in the periodontal and peri-implant tissues in the dog. J Clin Periodontol.

[2] Buser D, Weber HP, Donath K, Fiorellini JP, Paquette DW, Williams RC (1992). Soft tissue reactions to non-submerged unloaded titanium implants in beagle dogs. J Clin Periodontol.

[3] Misch CE, Dietsh-Misch F, Hoar J, Beck G, Hazen R, Misch CM (1999). A bone quality–based implant system: first year of prosthetic loading. J Oral Implantol.

[4] Barboza EP, Caúla AL, Carvalho WR (2002). Crestal bone loss around submerged and exposed unloaded dental implants: a radiographic and microbiological descriptive study. Implant Dent.

[5] Broggini N, McManus LM, Hermann JS, Medina R, Schenk RK, Buser D, Cochran DL (2006). Peri-implant inflammation defined by the implant-abutment interface. J Dent Res.

[6] Mishra PK, Wu W, Rozo C, Hallab NJ, Benevenia J, Gause WC (2011). Micrometer-sized titanium particles can induce potent Th2-type responses through TLR4-independent pathways. J Immunol.

[7] Canullo L, Micarelli C, Iannello G (2013). Microscopical and chemical surface characterization of the gingival portion and connection of an internal hexagon abutment before and after different technical stages of preparation. Clin Oral Implan Res.

[8] Sawase T, Wennerberg A, Hallgren C, Albrektsson T, Baba K (2000). Chemical and topographical surface analysis of five different implant abutments. Clin Oral Implan Res.

[9] Canullo L, Micarelli C, Lembo-Fazio L, Iannello G, Clementini M (2014). Microscopical and microbiologic characterization of customized titanium abutments after different cleaning procedures. Clin Oral Implan Res.

[10] Micarelli C, Canullo L, Baldissara P, Clementini M. Abutment screw reverse torque values before and after plasma cleaning. J Prosthodont. 2012. 10.11607/ijp.3396 23837162

[11] Gjermo P, Lyche Baastad K, Rölla G (1970). The plaque-inhibiting capacity of 11 antibacterial compounds. J Periodontal Res.

[12] Roberts WR, Addy M (1981). Comparison of the in vivo and in vitro antibacterial properties of antiseptic mouthrinses containing chlorhexidine, alexidine, cetyl pyridinium chloride and hexetidine: relevance to mode of action. J Clin Periodontol.

[13] Addy M (1986). Chlorhexidine compared with other locally delivered antimicrobials: a short review. J Clin Periodontol.

[14] Fine DH (1995). Chemical agents to prevent and regulate plaque development. Periodontol 2000.

[15] Rölla G, Melsen B (1975). On the mechanism of the plaque inhibition by chlorhexidine. J Dent Res.

[16] Yoon JG, Yoon J, Song JY, Yoon S, Lim CS, Seong H, Noh JY, Cheong HJ, Kim WJ. Clinical significance of a high SARS-CoV-2 viral load in the saliva. J Korean Med Sci. 2020;35(20). 10.3346/jkms.2020.35.e195 PMC7246183 32449329

[17] Gjermo P, Bonesvoll P, Rölla G (1974). Relationship between plaque-inhibiting effect and retention of chlorhexidine in the human oral cavity. Arch.

[18] Bonesvoll P, Lökken P, Rölla G, Paus PN (1974). Retention of chlorhexidine in the human oral cavity after mouth rinses. Arch.

[19] Kozlovsky A, Artzi Z, Moses O, Kamin-Belsky N, Greenstein RB (2006). Interaction of chlorhexidine with smooth and rough types of titanium surfaces. J Periodontol.

[20] Hjeljord LG, Rölla G, Bonesvoll P (1973). Chlorhexidine–protein interactions. J Periodontal Res.

[21] Jatzwauk L, Schöne H, Pietsch H (2001). How to improve instrument disinfection by ultrasound. J Hosp Infect.

[22] Bentley EM (1994). The value of ultrasonic cleaners in dental practice. Br Dent J.

[23] Canullo L, Penarrocha D, Micarelli C, Massidda O, Bazzoli M. Hard tissue response to argon plasma cleaning/sterilisation of customised titanium abutments versus 5-second steam cleaning: results of a 2-year post-loading follow-up from an explanatory randomised controlled trial in periodontally healthy patients. Eur J Oral Implantol. 2013;6(3). 24179979

[24] Canullo L, Peñarrocha D, Clementini M, Iannello G, Micarelli C (2015). Impact of plasma of argon cleaning treatment on implant abutments in patients with a history of periodontal disease and thin biotype: Radiographic results at 24-month follow-up of a rct. Clin Oral Implants Res.

[25] Bollen CM, Papaioanno W, Van Eldere J, Schepers E, Quirynen M, Van Steenberghe D (1996). The influence of abutment surface roughness on plaque accumulation and peri-implant mucositis. Clin Oral Implants Res.

[26] Kim YS, Shin SY, Moon SK, Yang SM (2015). Surface properties correlated with the human gingival fibroblasts attachment on various materials for implant abutments: a multiple regression analysis. Acta Odontol Scand.

[27] Gratton DG, Aquilino SA, Stanford CM (2001). Micromotion and dynamic fatigue properties of the dental implant–abutment interface. J Prosthet Dent.

[28] Tsuruta K, Ayukawa Y, Matsuzaki T, Kihara M, Koyano K (2018). The influence of implant–abutment connection on the screw loosening and microleakage. Int J Implant Dent.

[29] Bisognin ED, Harari ND, Machado SJ, da Silva CP, de Almeida Soares GD, Vidigal Jr GM. Evaluation of implant-abutment microgap and bacterial leakage in five external-hex implant systems: an in vitro study. Int J Oral Max Impl. 2012;27(2). 22442774

[30] Carr AB, Brunski JB, Labishak J, Bagley B (1993). Preload comparison between as-received and cast-to implant cylinders. J Dent Res.

[31] Alves DC, de Carvalho PS, Elias CN, Vedovatto E, Martinez EF (2016). In vitro analysis of the microbiological sealing of tapered implants after mechanical cycling. Clin Oral Invest.

[32] Olefjord I, Hansson S. Surface analysis of four dental implant systems. Int J Oral Max Impl. 1993;8(1). 8468084

[33] Lacey DC, De Kok B, Clanchy FI, Bailey MJ, Speed K, Haynes D, Graves SE, Hamilton JA (2009). Low dose metal particles can induce monocyte/macrophage survival. J Orthop Res.

[34] Yang SY, Zhang K, Bai L, Song Z, Yu H, McQueen DA, Wooley PH (2011). Polymethylmethacrylate and titanium alloy particles activate peripheral monocytes during periprosthetic inflammation and osteolysis. J Orthop Res.

[35] Geng D, Mao H, Wang J, Zhu X, Huang C, Chen L, Yang H, Xu Y (2011). Protective effects of COX-2 inhibitor on titanium-particle-induced inflammatory osteolysis via the down-regulation of RANK/RANKL. Acta Biomater.

[36] Jamal M, Shah M, Almarzooqi SH, Aber H, Khawaja S, El Abed R, Alkhatib Z, Samaranayake LP. Overview of transnational recommendations for COVID‐19 transmission control in dental care settings. Oral Dis. 2020. 10.1111/odi.13431 PMC7280672 32428372

[37] Wood NJ, Jenkinson HF, Davis SA, Mann S, O’Sullivan DJ, Barbour ME (2015). Chlorhexidine hexametaphosphate nanoparticles as a novel antimicrobial coating for dental implants. J Mater Sci Mater Med.

[38] Chin MY, Sandham A, De Vries J, van der Mei HC, Busscher HJ (2007). Biofilm formation on surface characterized micro-implants for skeletal anchorage in orthodontics. Biomaterials.

[39] Canullo L, Tallarico M, Chu S, Pennarocha D, Özcan M, Dent DM, Pesce P. Cleaning, disinfection, and sterilization protocols employed for customized implant abutments: an international survey of 100 universities worldwide. Int J Oral Max Impl. 2017;32(4). 10.11607/jomi.5402 28518179

